# Supplementary material for: Patterns of Intron Gain and Loss in Fungi
Source: PLoS Biol. 2004 Nov 30;2(12):e422. doi: 10.1371/journal.pbio.0020422 (PMC532390; doi:10.1371/journal.pbio.0020422)
Supplement: Table S1 — Also available at http://genes.mit.edu/NielsenEtAl/. (4.3 MB ZIP). [file pbio.0020422.st001.zip › NielsenEtAl/html/1218.html]

AN3459.1.NCU07153.1.MG00256.1.FG09893.1


```
 CLUSTAL W (1.82) Multiple Sequence Alignments - Introns Inserted


Sequence 1: MG00256.1	477 aa
Sequence 2: FG09893.1	476 aa
Sequence 3: NCU07153.1	476 aa
Sequence 4: AN3459.1	477 aa
Alignment Length: 477 aa
Number Identitical Residues: 311 aa
Alignment Score (without introns) 14490


MG00256.1 	MAPQLDSFFSQ~VDTLSDHFIER~LRAAVAIPSVSSDAARRPDVVR~MAHFLVDELKKLG
NCU07153.1	MAPQLDSYFQK~VDSLSDHFIDR~LRQAVAIPSISSEAARRPDVVR~MGQWLADELTKLG
FG09893.1 	MAPQLDGFFKQ~VDSSADHFIER~LRKAVAIPSISAEDARRPDVVR~MGEWLGDELKALG
AN3459.1  	MAPQLEPFFKQ2VDDLSGSFIDR1LRKAVAIPSVSAQDENRKDVFK0MAEFLASELKALG
          	*****: :*.: **  :. **:* ** ******:*::  .* **.: *..:* .**. **

MG00256.1 	AHVETRELGKQPDNPSLDLPPVVLGRYGNDPNKRTILVYGHYDVQPAELSDGWATEPFDL
NCU07153.1	ATVELRPLGKQ-EGTDLDLPPVVLARYGNDKNKRTILVYGHYDVQPAEKSDGWDTEPFDL
FG09893.1 	ASVELRPLGKQPHKEHLDLPPVVLARYGNDKNKRTILVYGHYDVQPAEKSDGWATEPFDL
AN3459.1  	AEVHQRPLGKQPGKEHLDLPPVVIARYGNDKNKKTILVYGHYDVQPALKEDGWATEPFEL
          	* *. * ****.    *******:.***** **:*************  .*** ****:*

MG00256.1 	TVGEDGRMFGRGSTDDKGPVLGWLNAIEAHQKAGVDFPVNLLMCFEGMEEYGSEGLDDLI
NCU07153.1	TVKEDGRMCGRGATDDKGPVLGWLNAIEAHKAAGIDFPVNLLMCFEGMEEYGSDGLEELV
FG09893.1 	TVDDKGRMFGRGSTDDKGPVLGWLNAIEAHQKAGVDFPVNLLMCFEGMEEYGSEGLDDLI
AN3459.1  	TVDDKGRMFGRGSTDDKGPVLGWLNVIDAHKKAGVELPVNLLCCFEGMEEYGSEGLEEFI
          	** :.*** ***:************.*:**: **:::***** **********:**::::

MG00256.1 	VAEAPKFFKDAEAVCISDNYWLGTEKPCLTYGLRGVNYYSIEVSGPGADLHSGVFGGTAQ
NCU07153.1	MAEGKKYFADADAVCISDNYWLGTERPCLTYGLRGCNYYSVEVSGPGADLHSGVFGGTAQ
FG09893.1 	KEEAKKYFADAEAVCISDNYWLGTEKPCLTYGLRGCNYYSVEISGPGADLHSGVFGGTAQ
AN3459.1  	HSESKGFFKDADAVCISDNYWLGTEKPCLTYGLRGCNYYSVSVSGPAQDLHSGVFGGSAH
          	  *.  :* **:*************:********* ****:.:***. *********:*:

MG00256.1 	EPMTDLVRVMGSLVDTDGNIQIPGVMEQVAPVTADEESLYDNISFTMDTLYESLGSKTVM
NCU07153.1	EPMTDLVRILASLVDTDGKIQIKGIAEQVAPVTPEEDGLYDDIAFTMETLHESLGSKTTI
FG09893.1 	EPMTDLVRVLGSLVNTGGEIQIPGIKEQVAPITVDEEGLYDGISFTMDNIHESLGSKTTI
AN3459.1  	EPMTDLISVLSKLVDSQGNILIPGIMDLVEPVTEEEKSLYTNISYTMENLHESLGSETGI
          	******: ::..**:: *:* * *: : * *:* :*..** .*::**:.::*****:* :

MG00256.1 	FDDKKKTLMRRWRYPSLSLHGIEGAFSAPGAKTVIPAKVIGKFSIRTVPNMEIDRTNEFV
NCU07153.1	FEDKKKTLMARWRFPSLSIHGVEGAFSNPGAKTVIPAKVIGKFSIRTVPDMEIEKTNQCV
FG09893.1 	FDDKKRTLMGRWRYPSLSIHGVEGAFSAPGAKTVIPAKVIGKFSIRTVPDMDIDKTNEAV
AN3459.1  	HPTKERTLMARWRFPSLSIHGIEGAYSAPGAKTVIPAKVIGKFSIRTVPNMESADVNRLV
          	.  *::*** ***:****:**:***:* *********************:*:   .*. *

MG00256.1 	YKYVNEQFTKLNSKNTLKVYAQHCGKWWVASPKHWNFSAAAKAVERVWGTAPDFTREGGS
NCU07153.1	YDHVNEVFKKLGSKNTMKVYAQHCGKWWKASPNHWNFAAAAKATERVWGMKPDFTREGGS
FG09893.1 	YKYVEDQFAKLGSKNTLKVYAQHTGKWWVASPKHWNFSAAAKAVERVWGVEPDYTREGGS
AN3459.1  	FDYIKSEFSKLNSKNTLDVWLQHDGKWWVASPKHWNFAAASKAVKQVFGVEPDMTREGGS
          	:.:::. * **.****:.*: ** **** ***:****:**:**.::*:*  ** ******

MG00256.1 	2IPVTLTFEQATGKNVLLLPMGSSTDGAHSINEKLDKRNYIEGIKLLGAYLHYVAEEPKQ
NCU07153.1	~IPITLTFEEATGKNVLLLPMGSSTDGAHSINEKLDKKNYIEGIKLLGAYLHYVAEEPMN
FG09893.1 	2IPVTLTFEEATGKNVLLLPMGSSTDGAHSINEKLDKRNYIEGIKLLGAYLHYVAEEPQT
AN3459.1  	2IPITLTFEEATGKNVLLLPMGSSTDAAHSVNEKLDKRNYIEGIKLLGAYLHYVAEEPMG
          	 **:*****:****************.***:******:********************  

MG00256.1 	E
NCU07153.1	A
FG09893.1 	-
AN3459.1  	A
          	
```
